# IL-33 at the Crossroads of Metabolic Disorders and Immunity

**DOI:** 10.3389/fendo.2019.00026

**Published:** 2019-01-30

**Authors:** Lei Tu, Lijing Yang

**Affiliations:** ^1^Division of Gastroenterology, Union Hospital, Tongji Medical College, Huazhong University of Science and Technology, Wuhan, China; ^2^Department of Radiation and Medical Oncology, Zhongnan Hospital, Wuhan University, Wuhan, China

**Keywords:** IL-33, metabolism, diabetes, innate & adaptive immune response, ST2

## Abstract

As a cytokine in interleukin-1(IL-1) family, interleukin-33(IL-33) usually exists in the cytoplasm and cell nucleus. When the cells are activated or damaged, IL-33 can be secreted into extracellular and regulate the functions of various immune cells through binding to its specific receptor suppression of tumorigenicity 2 (ST2). Except regulating the function of immune cells including T cells, B cells, dendritic cells (DCs), macrophages, mast cells, and innate lymphoid cells, IL-33 also plays an important role in metabolic diseases and has received an increasing attention. This review summarizes the regulation of IL-33 on different immune cells in lipid metabolism, which will help to understand the pathology of abnormal lipid metabolic diseases, such as atherosclerosis and type 2 diabetes.

## Introduction

IL-33, a new member of the IL-1 family, was discovered in 2005 (1) while its receptor ST2 containing intracellular domain Toll/IL-1R (TIR) was found in BALB/c-3t3 mouse fibroblasts in 1989 (1, 2). The receptor complex of IL-33 is composed of ST2 and interleukin-1 receptor accessory protein (IL-1RAcP). IL-33 mediates its biological effect through binding to its specific receptor ST2 (2, 3), whereas the expression of ST2 is restricted and determines the cellular responsiveness to IL-33 treatment (3). Two forms of ST2 have been demonstrated, a membrane-bound form (ST2L) and a soluble form (sST2), the latter which prevents its signaling as the decoy receptor for IL-33. IL-33 is mainly expressed in fibroblasts, epithelial cells and endothelial cells, and especially in high endothelial venules (HEV) (4). Indeed, as designated as an “alarmin,” IL-33 is usually released after cell injury to alert the immune system and initiate repair processes. In a recent study, islet mesenchymal-cell-derived IL-33 has been identified as an islet immunoregulatory feature (5). As the receptor of IL-33, ST2 is expressed in many immune cells. IL-33 is a dual-function cytokine. In the absence of inflammatory stimulation, IL-33 is located in the nucleus as a nuclear factor. Once the cell is damaged and/or necrotic, IL-33 can be released from the nucleus and then act as an endogenous “alarmin” (4). The activation signal produced by IL-33/ST2 pathway is transmitted to the cell and a series of signal transmissions activate nuclear factor kappa-light-chain-enhancer of activated B cells (NF-κB) and mitogen-activated protein kinase (MAPK) pathway to regulate immune response (1, 6). Under normal physiological condition, inflammation induced by a dysregulated lipid metabolism is benefit for the maintenance of homeostasis and is controlled to avoid excessive damage to the host. However, if not properly controlled, the inflammatory response will promote the excessive production of lipid metabolites, inflammatory cytokines and adhesion molecules, which lead to acute or chronic diseases (7), such as obesity, non-alcoholic steatohepatitis (NASH), atherosclerosis, and acute cardiovascular events. To date, an increasing body of evidence has demonstrated that IL-33 plays a critical role in the lipid metabolism. This review highlights the function of IL-33/ST2 axis on different immune cells in the metabolic disorders.

## IL-33/ST2L Signaling in Innate Immune Responses

IL-33 and ST2 have been shown to be expressed in human and murine adipose tissue, and IL-33 expression is strongly correlated with leptin expression in human adipose tissue (8). In addition, administration of IL-33 increases browning of white adipose tissue and energy expenditure in mice (9). These observations show that a critical role of IL-33 played in the adipose tissues homeostasis.

Macrophages have functional plasticity in adipose tissue inflammation, which can exhibit pro-inflammatory or anti-inflammatory function. According to the phenotypes and secreted cytokines, macrophages can be divided into two categories named as classical activated macrophages (CAM, M1 type) and alternatively activated macrophages (AAM, M2 type), respectively. CAM are generated in response to helper T1 cells (Th1 cells)-related cytokines, such as interferon-γ (IFN-γ) and tumor necrosis factor-α (TNF-α), while AAM polarization is linked to the helper T2 cells (Th2 cells)-related cytokines (IL-4 and IL-13) (10). Previous studies showed that AAM could attenuate adipose tissue inflammation and obesity-induced insulin resistance (11–14). It has been showed that ST2 can be detected on the cell surface of macrophages. IL-33 can promote the expression of lipopolysaccharide (LPS) receptor components such as myeloid differentiation factor 2 (MD2), toll-like receptor (TLR) 4, soluble cluster of differentiation 14 (CD14) and myeloid differentiation primary response gene 88 (Myd88), which result in an enhanced inflammatory cytokine production (15). However, IL-33 administration improves glucose tolerance, which is associated with the accumulation of M2 macrophages in adipose tissue of ob/ob mice that are the mutant mice to construct the model of Type II diabetes (16). As the result of purine metabolism disorder, gout is a very common metabolic disease in human (17, 18). Hyperlipidaemia is common in gout patients including increased low-density lipoprotein (LDL) cholesterol and decreased high-density lipoprotein (HDL) cholesterol (19). The serum IL-33 expression is predominantly increased in gout patients compared to healthy controls and positively correlated with the expression of HDL, while negatively correlated with LDL expression (20). It has been reported that the elevated IL-33 level is considerably reduced in renal impairment when compared with normal renal function in gout patients (20–22). These data suggest that IL-33 may prevent the kidney injury through regulating the lipid metabolism, which may be resulted from the AAM polarization.

Although ST2 can be detected on the cell surface of macrophages, IL-33/ST2 signaling cannot directly promote AAM polarization. The involvement of IL-33/ST2 signaling in the differentiation and activation of AAM is associated with type II cytokines induction (23–25). A previous finding showed that a population of cells expressing ST2 in adipose was potential to produce large amounts of Th2 cytokines in response to IL-33 (26). Recent studies have named this population as group 2 innate lymphoid cells (ILC2s), characterized by expressing ST2 receptor, and secreting type 2 cytokines such as IL-5 and IL-13 in response to IL-33 (27–30). In addition, soluble ST2 can prevent ILC2s from IL-33 stimulation (31). Recent observation has shown that ILC2s activation favors macrophages toward a protective AAM, which lead to a reduced lipid storage and decrease gene expression of lipid metabolism and adiposeness (32). Furthermore, it has showed that IL-13Rα2 may act as a critical checkpoint in the protective effect of the IL-33/IL-13 axis in obesity (33). In addition, IL-33 promotes β cell function through islet-resident ILC2s that elicite retinoic acid (RA)-producing capacities in macrophages and dendritic cells via the secretion of IL-13 and colony-stimulating factor 2 (5). These data suggest that IL-33 plays a protective role in the adipose tissue inflammation through regulating macrophage function, which is closely associated with the activation of ILC2 to produce type 2 cytokine and IL-4Rα signaling.

## IL-33/ST2L Signaling on T Cell Immune Responses

As a subset of T cells, the regulatory T cells (Tregs) play a critical role in suppressing autoimmune reactivity and have gained an increasing attention in the autoimmune diseases (34). It is shown that an impaired Tregs function is investigated in ST2 gene knockout mice with streptozotocin-induced diabetes, where the glycaemia and β cell loss are severe (35). Indeed, the exogenous IL-33 treatment propagates Tregs expressing the ST2 on the cellular surface, which suggests that the Tregs expansion induced by IL-33 administration is likely to be the result of a direct effect of IL-33 on ST2L^+^ Tregs (36, 37). Besides, ST2^+^ DCs stimulated by IL-33 to secrete IL-2, which promotes the selective expansion of ST2^+^ Tregs vs. non-Tregs, are required for *in vitro* and *in vivo* Tregs expansion (37, 38). In the Th1/Th17-mediated allograft rejection, IL-33 treatment can prevent allograft rejection through increasing ST2 positive Tregs in mice (39). In the mouse model of trinitrobenzene sulfonic acid (TNBS)-induced colitis, dextran sulfate sodium (DSS)-induced colitis or T cell adoptive transfer induced colitis, IL-33 can increase the number of Foxp3^+^ Tregs (40–42).

The Tregs also play a immunosuppressive function in obesity-associated inflammation (43). Interestingly, studies have also demonstrated that IL-33 maintain homeostasis in adipose tissue. A high level of ST2 expression is observed on human adipose tissue Tregs. Furthermore, IL-33 treatment can induce vigorous population expansion of Tregs in obese mice, and the changes of metabolic parameters are significantly correlated with the increased Tregs (44, 45). IL-33 signaling through the IL-33 receptor ST2 and the myeloid differentiation factor MyD88 pathway is essential for the development and maintenance of Tregs in visceral adipose tissue (44). However, ILC2-intrinsic IL-33 activation is required for Tregs accumulation *in vivo* and is independent of ILC2 type 2 cytokines but partially dependent on direct co-stimulatory interactions via the inducible costimulator ligand (ICOSL)/ICOS pathway (46). Concordantly, the ST2^+^ Tregs population is with a higher expression of activated marker ICOS and CD44 (38). Thus, IL-33 plays a protective role in adipose tissue inflammation through directly and indirectly regulating Tregs function.

It has also been reported that increasing severity of insulin resistance and microalbuminuria is strongly correlated with the decreased level of IL-33 in patients with diabetic nephropathy, where an enhanced Th1 and suppressed Th2 response is observed (47). ST2 is selectively and stably expressed on the surface of Th2 cells, and IL-33 can effectively induce the immune response of Th2 cells and the expression of Th2 related cytokines IL-5 and IL-13 without increasing IFN-γ expression (48, 49). These studies suggest that the ST2/IL-33 axis is closely associated with the Th1/Th2 response imbalance in the development of diabetes. Atherosclerosis is characterized by the formation of fibrotic plaques in the major arteries and increased Th1 immune response, which leads to myocardinfal iarction and stroke (50, 51). It has been shown that Th1-to-Th2 shift can reduce the development of atherosclerosis (52, 53). Due to the effect of IL-33 on Th2-type immune response, IL-33 exhibits a protective role in the pathogenesis of atherosclerosis (54). Previous findings also showed that the reduced level of IL-33 might increase the risk of atherosclerosis development for certain individuals (55). These data suggest a crucial role of IL-33 in the lipid metabolism through regulating T cells differentiation.

## Conclusion

**Figure 1:**
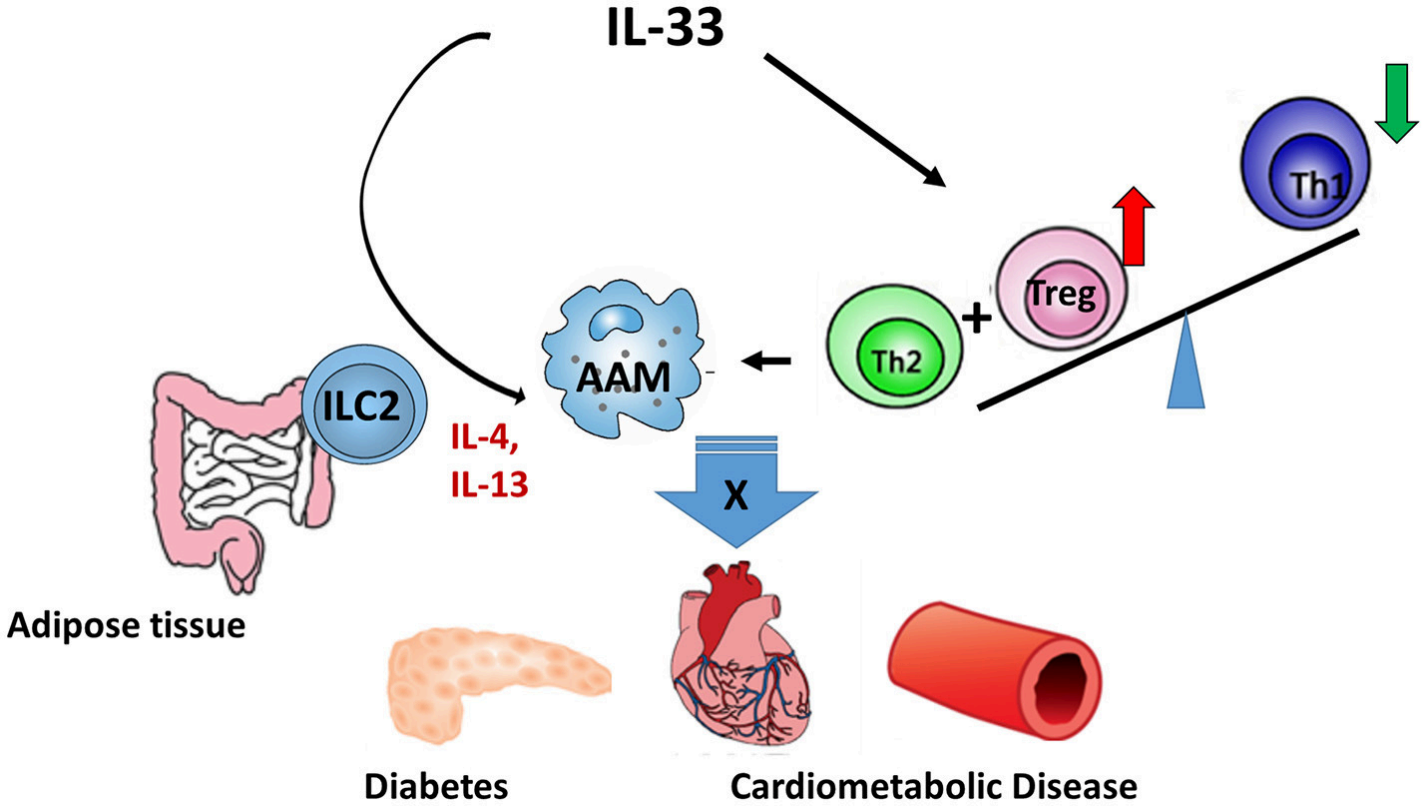
The regulatory role of IL-33 in metabolic diseases. IL-33/ST2 axis regulates metabolic diseases through: (1) promoting AAM polarization; (2) regulating the differentiation and functions of Treg and Th2; and (3) regulating the function of ILC2.

Due to the vital role of IL-33 in the metabolic homeostasis, a sound understanding of the production, regulation, and function of IL-33 will facilitate the treatment of metabolic disorders. The potential mechanisms (Figure 1) of IL-33/ST2 axis in the metabolic disorders may include: (1) IL-33 promotes the AAM polarization; (2) IL-33 regulates Tregs and Th2 differentiation and function; and (3) IL-33 regulates the function of ILC2. Notably, the AAM polarization induced by IL-33 depends on Type 2 cytokines, which may be released from ILC2. However, most studies in this area were mainly carried out on animal models and there were limited clinical trials. To what extent IL-33 contributes to metabolic disorders in humans still requires further investigation.

## Author Contributions

LT and LY reviewed the literature and wrote the first draft. LY finalized the manuscript. LT and LY have read and approved the final manuscript.

### Conflict of Interest Statement

The authors declare that the research was conducted in the absence of any commercial or financial relationships that could be construed as a potential conflict of interest.
